# Correlation between Occupational Stress and Coronary Heart Disease in Northwestern China: A Case Study of Xinjiang

**DOI:** 10.1155/2021/8127873

**Published:** 2021-04-20

**Authors:** Wu-Hong Lu, Wen-Qian Zhang, Fei Sun, Ya-Ting Gao, Yun-Juan Zhao, Ji-Wen Liu, Yi-Tong Ma, Muhuyati

**Affiliations:** ^1^Department of Medical Record Management, The First Affiliated Hospital of Xinjiang Medical University, Urumqi, 830054 Xinjiang, China; ^2^College of Public Health, Xinjiang Medical University, Urumqi, 830054 Xinjiang, China; ^3^Office of Medical Insurance Management, The Seventh Affiliated Hospital of Xinjiang Medical University, Urumqi, 830028 Xinjiang, China; ^4^Xinjiang Medical University, Urumqi, 830054 Xinjiang, China; ^5^Basic Medical College, Xinjiang Medical University, Urumqi, 830054 Xinjiang, China; ^6^Cardiovascular Center, The First Affiliated Hospital of Xinjiang Medical University, Urumqi, 830054 Xinjiang, China; ^7^State Key Laboratory of Pathogenesis, Prevention and Treatment of High Incidence Diseases in Central Asia, Heart Center, The First Affiliated Hospital of Xinjiang Medical University, Urumqi, 830054 Xinjiang, China

## Abstract

**Objective:**

To study the correlation between occupational stress and coronary heart disease in western China.

**Method:**

A case-control design was used. From June 2016 to May 2017, 310 patients with coronary heart disease (CHD) confirmed by coronary angiography (CAG) at the Heart Center of the First Affiliated Hospital of Xinjiang Medical University were recruited by cluster sampling, along with 536 healthy controls. The questionnaire was developed based on a Job Content Questionnaire (JCQ). An epidemiological survey was conducted to collect clinical data. Chi-squared test, analysis of variance, and binary logistic regression analysis were adopted.

**Results:**

(1) In the Han population, there were statistically significant differences in the composition of smoking, diets, sleep duration, sleep quality, and physical activity between two groups (all *P* < 0.05). In the Uygur population, statistically significant differences in the composition of smoking, drinking, diets, sleep quality, and physical activity were found between two groups (all *P* < 0.05). (2) Differences in sleep duration and physical activity between the Han and Uygur case groups were statistically significant (*P* < 0.05). (3) Differences in Gensini scores between the Han and Uygur case groups were statistically significant (*P* < 0.05). Differences in coronary artery lesions between the Han and Uygur case groups were statistically significant (*P* < 0.05). (4) In the Uygur population, the difference between the occupational stress level and CHD were statistically significant (*P* < 0.05). (5) The differences between the number of different pathological changes and the level of occupational stress in the Han and Uygur case groups were not statistically significant (*P* > 0.05). In the Han and Uygur case groups, the difference between the occupational stress level and Gensini high-level group were statistically significant (*P* < 0.05). (6) After adjustment for age and sex, significant increased risk effects for Han patients with CHD were found to be associated with sleep quality (OR = 1.88; 95% CI: 1.047-1.782; *P* < 0.05). Uygur patients with CHD was significantly associated with smoking (OR = 3.094; 95% CI: 1.025-1.103; *P* < 0.05) and occupation stress (OR = 1.523; 95% CI: 1.757-3.062; *P* < 0.05).

**Conclusion:**

Occupational stress is correlated with CHD for the Uygur population.

## 1. Introduction

Stress is prevalent in modern life, especially since occupational stress has been a mental health problem common to most people in modern society. Everyone living in the modern society has to face complex interpersonal relationship and stressful social environment every day. Occupational stress refers to the harmful psychological and physiological reaction that occurs when an individual's work ability and needs and his own resources cannot adapt to the work requirements [1]. It has been reported that occupational stress has become an important risk factor for decreased work ability, increasing absenteeism rate, and increasing incidence of occupational injury, as well as the primary potential cause or inducement factor of some diseases of workers [2].

In recent years, the incidence of coronary heart disease has increased significantly among the working population and is gradually becoming younger, while about 60%-80% of patients still need to stay at work [3]. Therefore, in addition to the traditional risk factors, occupational stress, as a potential risk factor for CHD, has attracted more and more attention from domestic and foreign scholars. After the standardization of occupational stress assessment methods, there are more and more researches in this field. Most studies suggest that occupational stress increases the risk of CHD. In 2015, the American Heart Association identified social factors as complementing traditional risk factors for cardiovascular diseases such as CHD [4].

Northwestern China is a multiethnic area; different ethnic groups have different living habits and eating styles, especially in Xinjiang. As an area with high incidence of cardiovascular diseases, it is urgent to explore the relationship between stress level of different occupational groups and CHD, so as to provide theoretical basis for prevention and treatment of CHD. The present study is aimed at exploring the correlation between occupational stress and coronary heart disease (CHD) in Xinjiang Han and Uygur population based on the JCQ scale.

## 2. Subjects and Methods

### 2.1. Subjects

This case-control study consecutively enrolled 310 unrelated consecutive CHD patients aged 18-70 years old, who were all admitted to The Heart Center of The First Affiliated Hospital of Xinjiang Medical University, The Heart Center, from January to December 2017.The diagnosis of CHD was based on the coronary artery angiography and the clinical manifestation.

Healthy subjects without a medical history of cardiovascular diseases were matched with CHD group in age and sex, and 536 subjects were selected as the control group after physical health examination in the hospital. All subjects enrolled in this study were employees aged over 18 years and had been working for more than 1 year and had no history of significant concomitant diseases, including cardiomyopathy, bleeding disorders, renal failure, thyroid disease, pulmonary hypertension, and malignancy.

This study protocol conformed to the Declaration of Helsinki and was approved by the Ethics Committee of Xinjiang Medical University, and a signed informed consent was obtained from all participants. All authors had no access to information that could identify individual participants after data collection.

### 2.2. Methods

#### 2.2.1. Questionnaire Survey

Occupational stress was evaluated by JCQ (Chinese version) [5]. JCQ includes 22 items, divided into three factors, job requirements, autonomy, and social support, and adopts Likert 4 rating. Each factor score is the sum of each factor entry; JCQ total score is the result of the sum of each factor score. The JCQ model takes social support as the auxiliary dimension and defines occupational tension with the work environment with high work requirements and low work autonomy. Low social support will aggravate occupational tension level. The *D*/*C* ratio is expressed as the ratio between the corrected work requirements and the work autonomy score based on the number of items. *D*/*C* ratio = work demand factor score/(autonomy factor score × 0.56); *D*/*C* ratio greater than 1 indicates high occupational stress. Less than 1 indicates low occupational stress. Based on this result, there were high occupational stress group and low occupational stress group in the JCQ mode. Studies have proved that the Chinese version of JCQ has good validity and reliability in a large number of surveys conducted among Chinese occupational population [6, 7].

#### 2.2.2. Diagnostic Criteria for CHD

The diagnostic criteria for CHD are in accordance with the diagnostic criteria for ischemic heart disease formulated by the world health organization. The patients with normal or less than 50% stenosis of all arteries were included. According to the results of coronary angiography, the score was calculated by the Gensini [8] scoring system, which included the degree of coronary artery stenosis and lesion location. In quantitative scoring, the score of a single vessel is the result of multiplying the basic score of the degree of stenosis of each coronary artery with the coefficient of the lesion. The integral summation of each lesion is the total Gensini integral. The lesions were classified into mild to moderate (1-14 points) and severe (≥14 points), as well as single, double, and multibranch groups according to the results of the total modified Gensini score.

### 2.3. Statistical Analysis

The data were statistically analyzed using SPSS18.0 software. The Hardy-Weinberg equilibrium law was used to detect the population representation of samples. Genotype and allele frequency count data were analyzed as (*n*, %) by chi-square test. Single-factor analysis was done first, and meaningful variables were included in the logistic regression model for further analysis. Binary logistic regression analysis was used to evaluate the influencing factors of CHD. Test level was *α* = 0.05.

## 3. Results

### 3.1. Comparison of Demographic Characteristics between the Case Group and Control Group

Among 846 subjects, 310 were CHD patients, including 202 Han nationality (65.2%) and 108 Uygur nationality (34.8%). 536 were healthy subjects, including 339 Han nationality (63.2%) and 197 Uygur nationality (36.8%).

No significant differences were found in terms of age, gender, ethnic group, education levels, marital status, and average monthly income between the case and control groups (all *P* > 0.05). It was suggested that the two groups have a good match in the demographic characteristics (Table 1).

### 3.2. Comparison of Living Habits between the Case Group and Control Group in Han and Uygur Populations

Table 2 shows comparison of living habits between the case group and control group in the Han and Uygur populations. In the Han population, there were statistically significant differences in the composition of smoking, diets, sleep duration, sleep quality, and physical activity between the two groups (all *P* < 0.05). In the Uygur population, statistically significant differences in the composition of smoking, drinking, diets, sleep quality, and physical activity were found between two groups (all *P* < 0.05). Differences in sleep duration and physical activity between the Han and Uygur case groups were statistically significant (*P* < 0.05; Table 3). For sleep duration, it is mainly 6-8 hours. In terms of physical activity, most Uygur patients do not exercise. It was suggested that there were ethnic differences in sleep duration, physical activity, and other living habits between Han and Uygur patients.

### 3.3. Comparison of Gensini Scores between Han and Uygur Case Groups

Table 4 shows the differences in Gensini scores between the Han and Uygur case groups were statistically significant (*P* < 0.05). The proportion of the high-level group of Uygur was higher. It was suggested that the coronary artery disease of Uygur was more serious than Han.

### 3.4. Comparison of Coronary Artery Lesions between Han and Uygur Case Groups

Differences in coronary artery lesions between the Han and Uygur case groups were statistically significant (*P* < 0.05; Table 5). The majority of single-vessel lesions were 48.0% and 50.0% in Han and Uygur, respectively.

### 3.5. The Relationship between Occupational Stress and CHD

Table 6 shows the comparison of occupational stress level and CHD between the Han and Uygur populations. In the Uygur population, the difference between the two groups were statistically significant (*P* < 0.05).

Table 7 shows the differences between the number of different pathological changes and the level of occupational stress in the Han and Uygur case groups were not statistically significant (*P* > 0.05). In the Han and Uygur case groups, the difference between the occupational stress level and Gensini high-level group were statistically significant (*P* < 0.05; Table 8). It was suggested that there were differences in occupational stress between the Han and Uygur patients with CHD.

### 3.6. Logistic Regression Analysis on the Risk Factors of CHD

Binary logistic regression analysis was used to evaluate the risk of CHD. After adjustment for age and sex, significant increased risk effects for Han patients with CHD were found to be associated with sleep quality (OR = 1.88; 95% CI: 0.747-1.082; *P* < 0.05) (Table 9). In addition, we noted that Uygur patients with CHD was significantly associated with smoking (OR = 3.094; 95% CI: 1.025-1.103; *P* < 0.05] and occupation stress (OR = 1.523; 95% CI: 1.757-3.062; *P* < 0.05) (Table 10).

## 4. Discussion

Cardiovascular and cerebrovascular diseases are known as “the first killer of human health.” The prevalence and mortality of cardiovascular diseases in China are in a rapid rise stage. Currently, it is clinically believed that CHD is closely related to genetic and environmental factors [9]. More and more people begin to pay attention to the potential relationship between occupational stress and CHD. It has been found that there was a significant relationship between occupational stress and CHD [10–12]. The study shows that in the Gensini high-level group, the proportion of the Uygur Gensini high-level group is higher than Han, and in the Uygur population, occupational stress is a risk factor of CHD, which may be due to the different lifestyle and eating habits with Han and increased risk of CHD in Uygur.

Some studies have shown that the level of Gensini score in the smoking group is significantly higher than that in the nonsmoking group [13]. Smoking is an independent risk factor for CHD, which is consistent with the results of this study. Long-term smoking can cause coronary artery vasodilation, coronary artery endothelial damage, coronary artery spasm, severity of stenosis, increase of platelet aggregation, von Willebrand factor, and aggravate the formation of coronary atherosclerotic plaque [14]. Some studies have shown that [15, 16], with the growth of age, the sleep quality of patients with CHD is worse; and patients are prone to stress, fear, and pessimism because they are worried about the condition, complications, prognosis, and long-term treatment, thus aggravating sleep disorders. And long-term and heavy treatment costs are not only the economic burden of the family but also may affect family harmony, making the patients social support lower, easy to cause sleep problems. The study suggest that sleep time and sleep quality are correlated with CHD. Logistic regression also shows that sleep quality is a risk factor of CHD in the Han population. The results of this study suggest that there is a relationship between CHD and physical exercise. Under the influence of the diet culture in Xinjiang, the intake of high-fat food is relatively large. No exercise will undoubtedly aggravate the occurrence and development of the disease. A large number of research data at home and abroad show that exercise can reduce the risk of CHD and related mortality [17]. Other studies have shown that even outside the control of traditional risk factors, 12.2% of global myocardial infarction is still related to lack of exercise [18].

Yang et al. [19] showed that the main pathological vessels of CHD in the 18-45-year-old groups were located in the anterior descending branch, and most of them were a single branch. Liu [20] showed that with the increase of age, the incidence of a single branch of coronary artery in patients with CHD gradually decreases, while the incidence of three branches of coronary artery was obviously increasing. However, Song et al. [21] showed that the age of the nonmilitary group was lighter than the military group, and the Gensini score was relatively high, but the results were not statistically significant. The study showed that the proportion of the high-level group of Uygur was higher. It was suggested that the coronary artery disease of Uygur was more serious than Han. And the differences in coronary artery lesions between the Han and Uygur case groups were statistically significant (*P* < 0.05).

Our study was limited in some ways. First, this was a case-control study that analyzed the correlations between occupational stress, behavioral features, and risk factors of CHD, through which no causal relationship could be determined. Second, since we collected data via a self-reported questionnaire, the possibility of underestimation or overestimation caused by reporting bias could not be excluded.

In conclusion, Xinjiang is a multiethnic gathering area. Different nationalities have different living habits and eating styles. As a high incidence area of cardiovascular disease, there is an urgent need to solve the pathogenesis of CHD in different ethnic groups. The results of this study not only find a new breakthrough but also provide guidance for the intervention of patients with CHD.

## Figures and Tables

**Table 1:** Comparison of general demographic characteristics between the case group and control group.

Variable	Control group (*n* = 536**)**	Case group (*n* = 310**)**	*χ* ^2^ value	*P* value
Age			2.040	0.361
≤40	62 (11.6)	28 (9.0)		
41-50	186 (34.7)	102 (32.9)		
≥51	288 (53.7)	180 (58.1)		
Gender			0.858	0.354
Male	416 (77.6)	249 (80.3)		
Female	120 (22.4)	61 (19.7)		
Ethnic group			0.312	0.576
Han	339 (63.2)	202 (65.2)		
Uygur	197 (36.8)	108 (34.8)		
Education levels			1.190	0.275
Junior college and below	242 (45.1)	152 (49.0)		
Undergraduate and above	294 (54.9)	158 (51.0)		
Marital status			0.260	0.878
Single	138 (25.7)	75 (24.2)		
Married	377 (70.4)	223 (71.9)		
Divorced or widowed	21 (3.9)	12 (3.9)		
Average monthly income			0.054	0.974
≤3000	64 (11.9)	37 (11.9)		
3100-5000	358 (66.8)	205 (66.2)		
≥5100	114 (21.3)	68 (21.9)		

**Table 2:** Comparison of living habits between the case group and control group in the Han and Uygur populations.

Variable	Han populations				Uygur populations			
	Control group (*n* = 339)	Case group (*n* = 202)	*χ* ^2^ value	*P* value	Control group (*n* = 197)	Case group (*n* = 108)	*χ* ^2^ value	*P* value
Smoking			153.038	<0.001			120.631	<0.001
Yes	94 (27.7)	167 (82.7)			27 (13.7)	83 (76.9)		
No	245 (72.3)	35 (17.3)			170 (86.3)	25 (23.1)		
Drinking			1.365	0.243			48.229	<0.001
Yes	202 (59.6)	110 (54.5)			24 (12.20)	52 (48.10)		
No	137 (40.4)	92 (45.5)			173 (87.80)	56 (51.90)		
Diets			6.628	0.036			6.532	0.038
Light	59 (17.4)	23 (11.4)			17 (8.60)	19 (17.60)		
General	213 (62.8)	123 (60.9)			129 (65.50)	58 (53.70)		
Heavy	67 (19.8)	56 (27.70)			51 (25.90)	31 (28.70)		
Sleep duration (h)			24.404	<0.001			3.84	0.147
<6	37 (10.9)	47 (23.3)			41 (20.8)	33 (30.6)		
6_-_8	279 (82.3)	128 (63.4)			145 (73.6)	71 (65.7)		
>8	23 (6.8)	27 (13.4)			11 (5.6)	4 (3.7)		
Sleep quality			12.756	0.002			18.328	<0.001
Poor	24 (7.1)	25 (12.4)			7 (3.6)	16 (14.8)		
General	216 (63.7)	143 (70.8)			178 (90.4)	78 (72.2)		
Good	99 (29.2)	34 (16.8)			12 (6.1)	14 (13.0)		
Physical activity			42.606	<0.001			21.785	<0.001
No	80 (23.6)	73 (36.1)			146 (74.1)	75 (69.4)		
Yes	103 (30.4)	17 (8.4)			20 (10.2)	2 (1.9)		

**Table 3:** Comparison of living habits between the Han and Uygur case groups.

Variable	Han populations (*n* = 202)	Uygur populations (*n* = 108)	*χ* ^2^ value	*P* value
Sleep duration (h)			8.081	0.018
<6	47 (23.3)	33 (30.6)		
6-8	128 (63.4)	71 (65.7)		
>8	27 (13.4)	4 (3.7)		
Physical activity			37.35	<0.001
No	73 (36.1)	75 (69.4)		
Yes	129 (63.9)	33 (30.6)		

**Table 4:** Comparison of Gensini scores between the Han and Uygur case groups.

Subgroup	Han populations (*n* = 202)	Uygur populations (*n* = 108)	*χ* ^2^ value	*P* value
Low-middle group	80 (39.6)	25 (23.1)	8.508	0.004
High-level group	122 (60.4)	83 (76.9)		

**Table 5:** Comparison of coronary artery lesions between the Han and Uygur case groups.

Subgroup	Han populations (*n* = 202)	Uygur populations (*n* = 108)	*χ ^2^* value	*P* value
Single-vessel disease	97 (48.0)	54 (50.0)	10.792	0.005
Double-vessel disease	73 (36.1)	23 (21.3)		
Triple-vessel disease	32 (15.9)	31 (28.7)		

**Table 6:** The comparison of between occupational stress level and CHD in different ethnic groups.

Subgroup	Han populations				Uygur populations			
	Control group (*n* = 339)	Case group (*n* = 202)	*χ* ^2^	*P* value	Control group (*n* = 197)	Case group (*n* = 108)	*χ* ^2^	*P* value
High occupational stress	103 (30.4)	65 (32.2)	0.19	0.663	55 (27.9)	44 (40.7)	5.231	<0.001
Low occupational stress	236 (69.6)	137 (67.8)			142 (72.1)	64 (59.3)		

**Table 7:** Comparison of the number of pathological changes and occupational stress levels between the Han and Uygur case groups.

Subgroup	Han populations					Uygur populations				
	Single-vessel disease	Double-vessel disease	Triple-vessel disease	*χ* ^2^	*P*	Single-vessel disease	Double-vessel disease	Triple-vessel disease	*χ* ^2^	*P*
High occupational stress	36 (37.1)	19 (26.0)	10 (31.3)	2.361	0.307	22 (40.7)	8 (34.8)	14 (45.2)	0.589	0.745
Low occupational stress	61 (62.9)	54 (74.0)	22 (68.7)			32 (59.3)	15 (65.2)	17 (54.8)		

**Table 8:** Comparison of the occupational stress levels and different Gensini groups between the Han and Uygur case groups.

Subgroup	Low-middle group				High-level group			
	Han populations	Uygur populations	*χ* ^2^	*P*	Han populations	Uygur populations	*χ* ^2^	*P*
High occupational stress	26 (32.5)	5 (20.0)	1.43	0.232	39 (50.0)	83 (65.4)	4.728	<0.001
Low occupational stress	54 (67.5)	20 (80.0)			39 (50.0)	44 (34.6)		

**Table 9:** Logistic regression analysis on the risk factors of CHD in the Han population.

Influencing factor	*B* value	*S*_*b*_	Wald	*P*	OR	95% CI
			*χ* ^2^			
Smoking	-0.234	0.244	1.017	0.325	0.107	0.535~1.158
Diets	-0.239	0.197	1.474	0.225	0.787	0.638~1.648
Sleep duration	0.025	0.242	0.011	0.918	1.025	1.213~2.914
Sleep quality	0.631	0.224	7.965	<0.05	1.88	1.047~1.782
Physical activity	-0.106	0.094	1.267	0.26	0.899	0.766~2.049
Occupational stress	0.226	0.251	0.81	0.368	1.253	0.679~0.999

**Table 10:** Logistic regression analysis on the risk factors of CHD in the Uygur population.

Influencing factor	*B* value	*S*_*b*_	Wald	*P*	OR	95% CI
			*χ* ^2^			
Smoking	2.989	0.365	17.034	<0.05	3.094	1.025~1.103
Diets	0.403	0.285	2.003	0.157	1.497	0.856~2.616
Sleep duration	0.506	0.354	2.048	0.152	1.659	0.829~3.317
Sleep quality	0.319	0.431	0.547	0.46	1.375	0.591~3.202
Physical activity	0.174	0.167	0.196	0.658	0.929	0.67~1.288
Occupational stress	0.42	0.357	21.39	<0.05	1.523	1.757~3.062

## Data Availability

No data were used to support this study.
